# Incidence of chronic disease following smoking cessation treatment: A matched cohort study using linked administrative healthcare data in Ontario, Canada

**DOI:** 10.1371/journal.pone.0288759

**Published:** 2023-07-26

**Authors:** Dolly Baliunas, Sabrina Voci, Peter Selby, Claire de Oliveira, Paul Kurdyak, Laura Rosella, Laurie Zawertailo, Longdi Fu, Rinku Sutradhar

**Affiliations:** 1 School of Health and Medical Sciences, University of Southern Queensland, Ipswich, Queensland, Australia; 2 Clinical Research – Addictions, Centre for Addiction and Mental Health, Toronto, Ontario, Canada; 3 Dalla Lana School of Public Health, University of Toronto, Toronto, Ontario, Canada; 4 School of Public Health, University of Queensland, Herston, Queensland, Australia; 5 Nicotine Dependence Service, INTREPID Lab, Addictions Program, Centre for Addiction and Mental Health, Toronto, Ontario, Canada; 6 Institute for Mental Health Policy Research, Centre for Addiction and Mental Health, Toronto, Ontario, Canada; 7 Department of Family and Community Medicine, University of Toronto, Toronto, Ontario, Canada; 8 Department of Psychiatry, University of Toronto, Toronto, Ontario, Canada; 9 Centre for Health Economics and Hull York Medical School, University of York, Heslington, York, United Kingdom; 10 Institute of Health Policy, Management and Evaluation, University of Toronto, Toronto, Ontario, Canada; 11 ICES, Toronto, Ontario, Canada; 12 Centre for Addiction and Mental Health, Toronto, Ontario, Canada; 13 Department of Pharmacology and Toxicology, University of Toronto, Toronto, Ontario, Canada; University of Botswana School of Medicine, BOTSWANA

## Abstract

Scarce evidence is available on the impact of real-world smoking cessation treatment on subsequent health outcomes, such as incidence of chronic disease. This study compared two cohorts of people that smoke—those that enrolled in a smoking cessation program, and a matched control that had not accessed the program—to assess the incidence of cancer, chronic obstructive pulmonary disease, diabetes, hypertension, and major cardiovascular events over a 5-year follow-up period. We selected five sub-cohorts with matched treatment-control pairs in which both individuals were at risk of the five chronic diseases. Incident chronic disease from index date until December 31, 2017, was determined through linkage with routinely collected healthcare data. The cumulative incidence of each chronic disease was estimated using the cumulative incidence function with death as a competing risk. Gray’s test was used to test for a difference between matched treatment and control groups in the chronic disease-specific cumulative incidence function over follow-up. Analyses were stratified by sex. Among females, cumulative incidence of diabetes was higher over follow-up for the treatment group (5-year cumulative incidence 5.8% vs 4.2%, p = 0.004), but did not differ for the four other chronic diseases. Among males, cumulative incidence of chronic obstructive pulmonary disease (12.2% vs 9.1%, p < 0.001) and diabetes (6.7% vs 4.8%, p < 0.001) both had higher 5-year cumulative incidence for the treated versus control groups but did not differ for the other three chronic diseases. We conclude that accessing primary-care based smoking cessation treatment is associated with increased incidence of diabetes for both sexes, and chronic obstructive pulmonary disease for males (possibly due to under diagnosis prior to treatment), within 5 years of treatment. The associations detected require further research to understand causal relationships.

## Introduction

Tobacco smoking harms nearly every organ in the body and is a leading cause of preventable morbidity and premature mortality [1]. Individuals who smoke lose at least a decade of life expectancy compared to those who have never smoked [2]; major causes of this excess mortality include cancer as well as vascular and respiratory disease [1, 2]. Quitting smoking greatly reduces the risk of developing smoking-related diseases including cardiovascular disease, several types of cancer, and chronic obstructive pulmonary disease (COPD) [3]. The extent of risk reduction, and timeline over which it occurs, varies between diseases. Risk of cardiovascular events is reduced substantially within five years, and after 15 years of quitting the risk is close to that of individuals who have never smoked [4, 5]. Approximately 10–15 years after smoking cessation, lung cancer risk decreases to half that of those who continue to smoke and continues to decline as time since cessation increases [3]. While smoking cessation can prevent the development of COPD and attenuate disease progression [6], lost lung function already present at the time of smoking cessation is not fully recovered [7]. In addition to cessation itself, reducing the number of cigarettes per day may be associated with some health benefits among those who continue to smoke. A meta-analysis of people who smoke heavily (followed from 5 to 40 years) found that those who reduced their smoking lowered their lung cancer risk, but not their risk of all cancers or all smoking-related cancers [8]. The risk of cardiovascular disease was also lowered among those who reduced from heavy to light smoking [9], but was not lowered by a 50% reduction in cigarettes per day [8, 10, 11].

Although smoking cessation has the greatest impact on reducing the health risks associated with smoking, it is important to also study the health benefits associated with receiving smoking cessation treatments in real-world settings [12], and not just smoking cessation. This is because, in part, with only 3–5% of untreated quit attempts achieving abstinence [13], interventions that result in 5–10% abstinence may be considered effective [14], and most patients presenting for treatment will not achieve sustained abstinence. Furthermore, the focus on abrupt abstinence is too narrow to encompass the breadth of treatment trajectories and outcomes that occur. Individuals seeking treatment may relapse and transition between smoking and non-smoking repeatedly, as many make several failed attempts to quit before they finally succeed [15–17]. In addition, some individuals presenting for treatment will set a goal to reduce, but not stop, smoking, or will adopt a reduce-to-quit strategy, whereby they gradually reduce cigarette consumption as a cessation strategy [18–21]. An awareness of the health outcomes associated with smoking cessation treatment can help guide healthcare decision makers and clinicians treating these patients, such as deciding whether and when to provide screening or preventive treatment for particular conditions.

Aside from studies conducted to detect adverse events associated with cessation medications (nicotine replacement therapy (NRT), varenicline, bupropion) [22, 23], few studies have examined incident health outcomes following smoking cessation treatment. Where published, existing studies have examined cause-specific hospitalizations or re-hospitalizations. For example, among Massachusetts Medicaid enrolees, use of a smoking cessation pharmacotherapy insurance benefit was associated with a significant decrease in hospitalization for acute myocardial infarction and other acute coronary heart disease diagnoses, but no significant change was observed in hospitalizations for respiratory diagnoses [24]. In another study, re-hospitalization and mortality outcomes were examined among people who smoke and initiated smoking cessation treatment while in hospital; these patients experienced significantly lower rates of smoking-related readmissions compared to patients who smoke and received usual care [25]. The Lung Health Study compared the health outcomes of people with prevalent COPD who either received cessation treatment, consisting of NRT and behavioural support, or usual care [26]; after 5 years, there was no significant difference in lung cancer or hospitalizations for respiratory disease between the usual care and smoking cessation treatment groups, although there was greater smoking cessation and a slower decline in lung function among those who received smoking cessation treatment.

This dearth of research examining the incidence of chronic disease following smoking cessation treatment indicates a gap in the literature. Data collected during routine interactions with the healthcare system provides an opportunity to address this gap. Such data, from actual patients who have received smoking cessation treatment in typical clinical settings and conditions, can allow us to assess whether the healthcare outcomes we hope to achieve are ultimately being realized in the real world. Therefore, the objective of the current study was to compare the incidence of cancer, COPD, diabetes, hypertension, and major cardiovascular events during a 5-year follow-up period for people who smoke and had accessed a smoking cessation treatment program, versus people who smoke and had not accessed the program, in Ontario, Canada.

## Materials and methods

### Study design

We conducted a retrospective matched cohort study to compare the risk of developing chronic disease among individuals who had enrolled in the Smoking Treatment for Ontario Patients (STOP) program against matched control Ontarians who smoke but had not accessed the program. Incident chronic disease was determined through linkage with health administrative data. This study was approved by the Research Ethics Board of the Centre for Addiction and Mental Health (#110–2019) and adheres to the Reporting of Studies Conducted Using Observational Routinely-Collected Health Data (RECORD) guidelines (see S1 Checklist) [27].

### Smoking cessation treatment

The STOP program delivers smoking cessation treatment to patients at partnering healthcare organizations across the province of Ontario. Prior to receiving treatment, patients provide written informed consent. Patients are eligible to receive up to 26 weeks of NRT within a 12-month period, and behavioural counselling delivered by healthcare practitioners trained in smoking cessation interventions. Although treatment is tailored to individual need and can vary, a majority of patients receive a combination of transdermal patch plus a single form of short-acting NRT (e.g., gum, inhaler) [28]. Further description of the STOP program is provided elsewhere [29].

### Matched cohort creation

In a parent study, we derived a treatment cohort who sought smoking cessation treatment via the STOP program and a matched control cohort who smoked but had not accessed the program (detailed selection criteria for participants that made up these cohorts can be found in the parent study or S1 Fig) [29]. The parent study treatment cohort consisted of patients who had enrolled in the STOP program between 1 July 2011 and 31 December 2012. The matched control cohort was formed using the 2007/2008, 2009/2010, and 2011/2012 cycles of the Canadian Community Health Survey (CCHS), a cross-sectional population-based survey that collects self-reported health-related data including smoking behaviours; detailed survey methodology is reported elsewhere [30]. If a CCHS respondent appeared in multiple cycles, only data from their most recent survey was retained in order to better align with the enrolment timeframe for the treatment cohort. Index date was the date of enrolment in the STOP program for the treatment cohort and the date of CCHS survey completion for the control cohort. Each treated individual was matched to one control individual using a combination of hard matching (sex and age ± 2 years at index date) and propensity-score matching (using a greedy algorithm without replacement with a caliper width of 0.2 standard deviations of the estimated propensity score logit). The propensity score estimated the probability of treatment for each individual. This was done using multivariable logistic regression with the following baseline variables: age at index, education, household income, number of cigarettes smoked per day, age first started smoking, comorbidity burden (determined by Aggregated Diagnostic Groups [31]) and the rate of emergency department visits and hospitalizations in the two years prior to index date. Further details of the matching process used in the parent study are described elsewhere [29].

For the current study, we selected participants from the population of matched treatment-control cohorts that were derived previously. We identified five matched sub-cohorts from the parent study (one matched sub-cohort for each of the five chronic disease outcomes). These sub-cohorts were at risk of: (i) cancer, (ii) COPD, (iii) diabetes, (iv) hypertension, or (v) a major cardiovascular event. Individuals were deemed to be “at risk of” each outcome if there was no known record of having experienced the outcome. For each sub-cohort, individuals who had already experienced the disease outcome at index were excluded because they were no longer at risk of incident disease after index; we also excluded the individual with whom they had been matched in the parent study to preserve the 1:1 matched design. Thus, for each chronic disease, we retained previously matched treatment-control pairs who were both at risk for developing the disease outcome after index. For a flow chart illustrating derivation of the at-risk matched sub-cohorts, see S1 Fig.

### Outcomes

The primary outcomes were incidence of cancer, COPD, diabetes, hypertension, and major cardiovascular events (i.e., acute myocardial infarction, stroke, percutaneous coronary intervention, coronary artery bypass graft, or death from ischaemic heart disease or cerebrovascular disease) from index date until 31 December 2017, loss of Ontario Health Insurance Plan eligibility (OHIP; this plan provides jurisdiction-wide health coverage), or death, whichever occurred first.

### Data sources

Data from the STOP program and the CCHS were previously linked to health administrative data sets. All datasets were linked using unique encoded identifiers and analyzed at ICES [32]. ICES is an independent, non-profit research institute whose legal status under Ontario’s health information privacy law allows it to collect and analyze healthcare and demographic data, without consent, for health system evaluation and improvement.

### STOP baseline assessment or CCHS survey

Descriptive characteristics pertaining to smoking history were self-reported in the STOP baseline assessment questionnaire or the CCHS: smoking frequency, number of cigarettes smoked per day, and age when first tried smoking. Duration of smoking was calculated by subtracting age when first tried smoking from age at index.

### ICES datasets

Derivation of at-risk sub-cohorts, and ascertainment of chronic disease outcomes, were achieved using several datasets. These datasets were checked at index to identify people at risk of each health outcome, and over the follow-up period to ascertain if, and when, a health outcome had occurred. Diabetes, COPD, and hypertension were identified based on existing chronic condition datasets derived by ICES: the Ontario Diabetes Dataset [33], Ontario Chronic Obstructive Pulmonary Disease database [34], and Ontario Hypertension Dataset [35]. ICES applies validated algorithms to the healthcare administrative data of all Ontarians on a recurring basis to identify patients with these medical conditions. Cancer diagnoses were identified from the Ontario Cancer Registry [36], which collects data on Ontario residents newly diagnosed with cancer (except for basal cell carcinoma and squamous cell carcinoma of the skin) or who have died of cancer. Major cardiovascular events were defined as hospital admissions or emergency department (ED) visits for acute myocardial infarction, stroke, percutaneous coronary intervention, coronary artery bypass graft, or death from ischaemic heart disease or cerebrovascular disease [37]. These events were ascertained from the Canadian Institute for Health Information Discharge Abstract Database (DAD), National Ambulatory Care Reporting System (NACRS), and Office of the Registrar General Vital Statistics–Deaths database.

Additional datasets were used to identify baseline characteristics. Age at index, sex and postal code were obtained from the Registered Persons Database. Immigration category was obtained from the Immigration, Refugee and Citizenship Canada Permanent Resident database [38]. Postal code was linked to census data to obtain neighbourhood-level socioeconomic indicators (household income, employment and educational attainment quintiles) and the Rurality Index of Ontario (RIO 2008) [39] scores to assess rurality of residence. In addition to the chronic disease datasets used to define the at-risk cohorts and ascertain outcomes, the following datasets were used to assess comorbidities at baseline: asthma (Ontario Asthma Dataset [40]), congestive heart failure (Congestive Heart Failure Dataset [41]) and myocardial infarction (Ontario Myocardial Infarction Dataset [42]) at index. Comorbidity burden was determined using The John Hopkins ACG^®^ System (Version 10) Aggregated Diagnosis Groups (ADGs); scores were calculated using a two-year lookback window from index date and categorized into four groups (0, 5, 6–9, 10+), with higher scores indicating greater comorbidity burden [31]. Healthcare utilization in the two years prior to index date was ascertained using data on outpatient physician visits (for any reason) from the OHIP database, hospitalizations from the DAD and Ontario Mental Health Reporting System, and emergency department visits from the NACRS. Occurrence and date of any deaths were identified using the Registered Persons Database. Causes of death were ascertained from the Office of the Registrar General Vital Statistics–Deaths database. Further details about baseline measures and data sources are provided elsewhere [29, 32].

### Statistical analyses

Baseline characteristics for each of the matched sub-cohorts were described using frequencies and percentages for categorical measures and mean and standard deviation for continuous measures. Standardized mean differences (SMD) were computed to examine balance in the distributions of baseline characteristics between the treatment and control groups; an SMD > 0.1 was considered a meaningful imbalance.

Within each matched sub-cohort, the risk of chronic disease was estimated for treatment and control groups using the cumulative incidence function approach, where death (occurring prior to chronic disease) was treated as a competing event. Individuals whose observation terminated due to study end were considered right-censored at that time. Under this approach, the estimated 5-year risk of chronic disease was reported for treatment and control groups. Gray’s test was used to determine if the risk of chronic disease over time statistically differed between treatment and control groups.

All analyses were stratified by sex. As sex was a hard-matched variable, all stratified analyses could be done without breaking matched pairs. A p value less than 0.05 was considered statistically significant. There were no missing data. Analyses were conducted using SAS Enterprise Guide version 7.12 software.

## Results

### Cancer

The treatment and control groups in the matched sub-cohort at risk for cancer were well-balanced at index on all sociodemographic characteristics, including age, sex, education, employment, rurality/neighbourhood income and migrant status. Some imbalances remained on smoking characteristics, prevalent comorbidities and healthcare utilization in the two years prior to index. There was a higher proportion of those who smoked daily and a lower proportion who smoked occasionally in the treatment versus control group (both sexes). In addition, the number of outpatient visits and the proportion of those having at least one ED visit were also higher in the treatment group versus the matched control group (males only). The above was true for all five at-risk matched sub-cohorts.

The following additional imbalances were found between the treatment and control groups in the matched sub-cohort at risk for cancer. Three prevalent health conditions were more common in the treatment group versus control group: COPD (females: 29.5% vs. 19.8%; males: 26.5% vs. 17.3%), diabetes (males: 16.2% vs. 11.4%) and asthma (females: 24.3% vs. 19.0%). The proportion having made an outpatient visit two years prior to index was also higher in the treated versus controls (females: 96.6% vs. 93.7%; males: 93.9% vs. 83.7%). Baseline characteristics of the treatment and control groups in the matched sub-cohort at risk for cancer, stratified by sex, are described in S1 Table.

**Fig 1:**
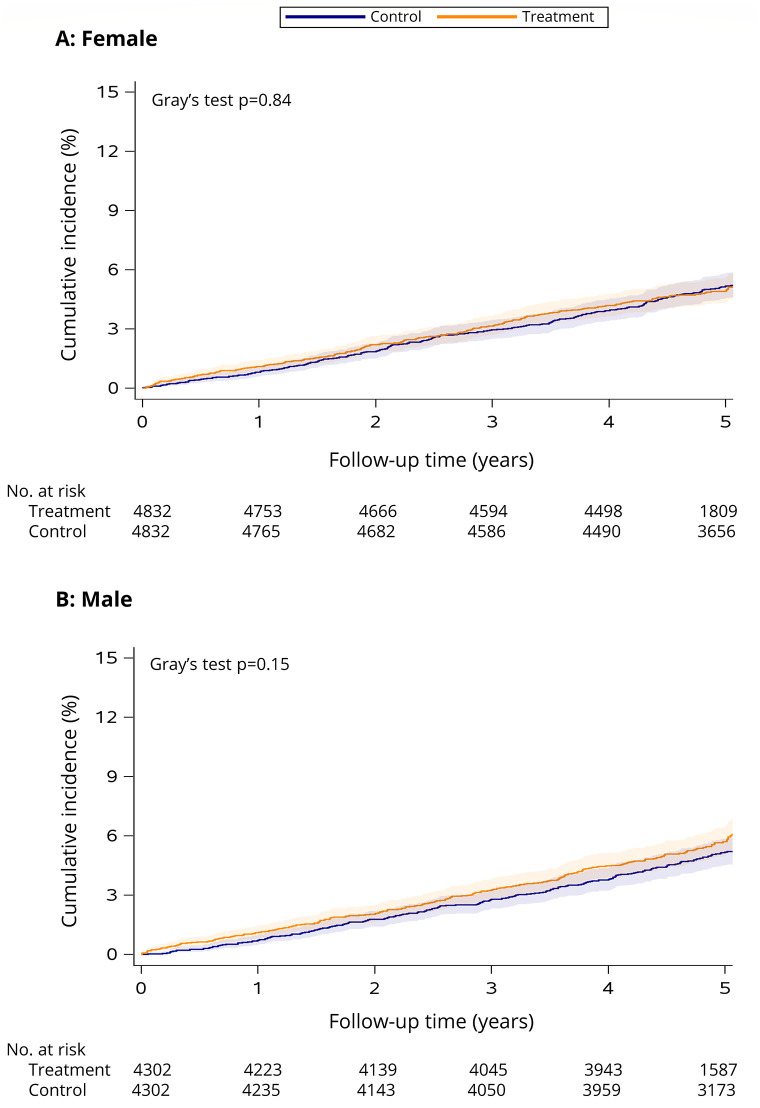
Cumulative incidence of cancer among female (A) and male (B) smoking cessation treatment patients versus matched controls. The follow-up period begins the day after enrolment in smoking cessation treatment (treatment cohort) or survey completion (control cohort) in 2011/2012 and ends December 31, 2017 (or date of death if it occurred first). Shaded areas indicate the 95% CI. Number of individuals at risk at each time point is presented below the *x* axis.

See Table 1 for the estimated incidence of cancer within 5 years of follow-up in the treatment and control groups, accounting for right-censoring and treating death as a competing risk. The cumulative incidence of cancer did not differ significantly between treatment and control groups, among both sexes (females: Gray’s test *p* = 0.84, see Fig 1A; males: Gray’s test *p* = 0.84, see Fig 1B).

**Table 1:** Estimated risk of chronic disease among treated and matched control groups at 5 years post index, accounting for death as a competing event.

	Females		Males	
	N	Cumulative incidence of disease outcome at 5 years (95% CI)	N	Cumulative incidence of disease outcome at 5 years (95% CI)
**Cancer**				
Treatment	4,832	4.9% (4.3%–5.6%)	4,302	5.7% (5.0%–6.5%)
Control	4,832	5.2% (4.5%–5.8%)	4,302	5.2% (4.5%–5.9%)
**COPD**				
Treatment	3,024	11.0% (9.9%–12.2%)	2,881	12.3% (11.0%–13.6%)
Control	3,024	10.7% (9.6%–11.8%)	2,881	9.1% (8.1%–10.2%)
**Diabetes**				
Treatment	4,074	5.8% (5.1%–6.5%)	3,469	6.8% (6.0%–7.8%)
Control	4,074	4.3% (3.7%–5.0%)	3,469	4.7% (4.0%–5.4%)
**Hypertension**				
Treatment	3,000	8.9% (7.9%–10.0%)	2,577	10.4% (9.1%–11.6%)
Control	3,000	7.9% (7.0%–8.9%)	2,577	10.0% (8.9%–11.2%)
**Major CV events**				
Treatment	5,007	4.5% (3.9%–5.1%)	4,272	6.9% (6.2%–7.8%)
Control	5,007	4.5% (3.9%–5.1%)	4,272	6.4% (5.6%–7.1%)

Abbreviations: COPD = chronic obstructive pulmonary disease; CV = cardiovascular; IQR = interquartile range; SD = standard deviation.

### COPD

In addition to the common imbalances described above (first paragraph of Results), there were further imbalances between the treatment and control groups in the matched sub-cohort at risk of COPD. The treatment group smoked more cigarettes per day than the control group (females: 15.48 vs. 14.42). Compared to the control group, the treatment group had a higher prevalence of diabetes at index (males: 12.6% vs. 8.4%) and a higher proportion had at least one outpatient visit (males: 92.1% vs. 80.8%). There was also a higher proportion with 0–5 ADG comorbidities in the treatment versus control group (females: 51.2% vs. 44.7%). Thus, findings suggest somewhat higher comorbidity and healthcare utilization among treated males versus control males at risk of COPD, but lower overall comorbidity burden among treated females versus control females. Baseline characteristics of the matched sub-cohort at risk of COPD, stratified by sex, are described in S2 Table.

**Fig 2:**
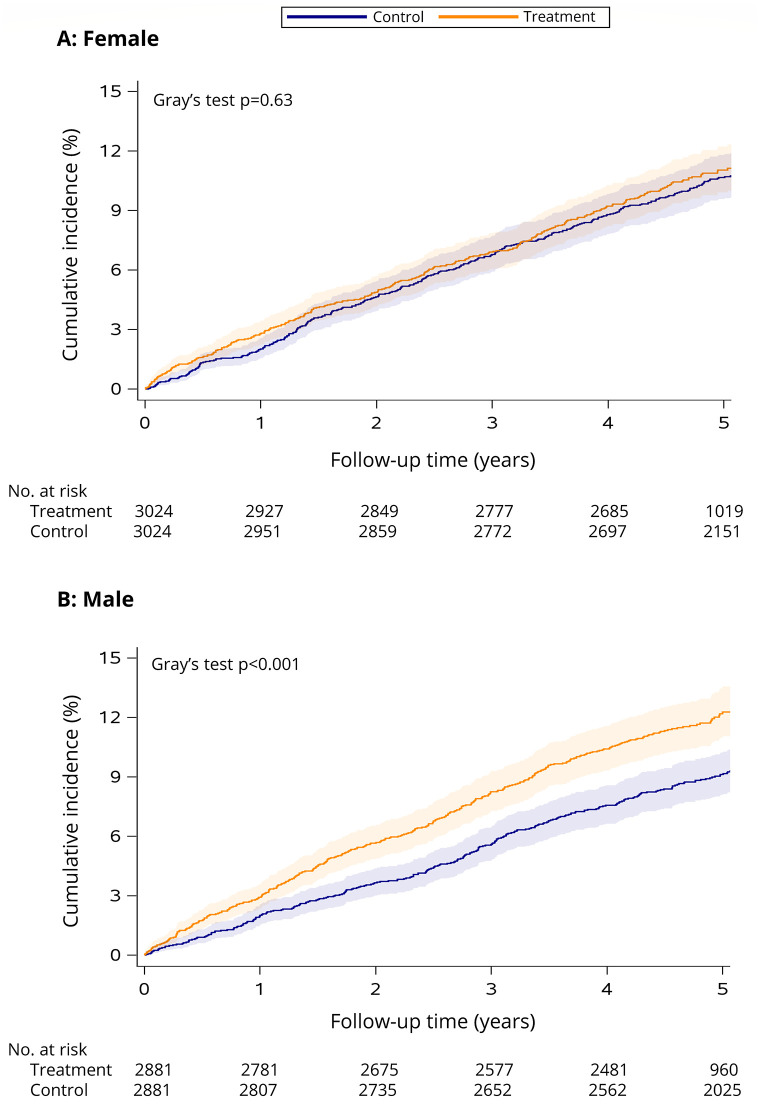
Cumulative incidence of COPD among female (A) and male (B) smoking cessation treatment patients versus matched controls. The follow-up period begins the day after enrolment in smoking cessation treatment (treatment cohort) or survey completion (control cohort) in 2011/2012 and ends December 31, 2017 (or date of death if it occurred first). Shaded areas indicate the 95% CI. Number of individuals at risk at each time point is presented below the *x* axis.

See Table 1 for the estimated incidence of COPD within 5 years of follow-up in the treatment and control groups. While the cumulative incidence of COPD did not differ significantly between treated and control females (Gray’s test *p* = 0.63, see Fig 2A), it was significantly higher over time for treated males (Gray’s test *p* < 0.001, see Fig 2B).

### Diabetes

In addition to the common imbalances described above (first paragraph of Results), there were further imbalances between the treatment and control groups in the matched sub-cohort at risk of diabetes. Two prevalent health conditions were more common in the treatment group: COPD (females: 27.3% vs. 17.6%; males: 24.5% vs. 15.0%) and asthma (females: 23.3% vs. 18.0%). The proportion having at least one outpatient visit in the two years prior to index was higher in the treatment versus control group (females: 96.3% vs. 93.5%; males: 93.2% vs. 82.1%), as was the proportion who had been hospitalized (males:13.5% vs. 10.1%). Baseline characteristics of the matched sub-cohort at risk of diabetes, stratified by sex, are described in S3 Table.

**Fig 3:**
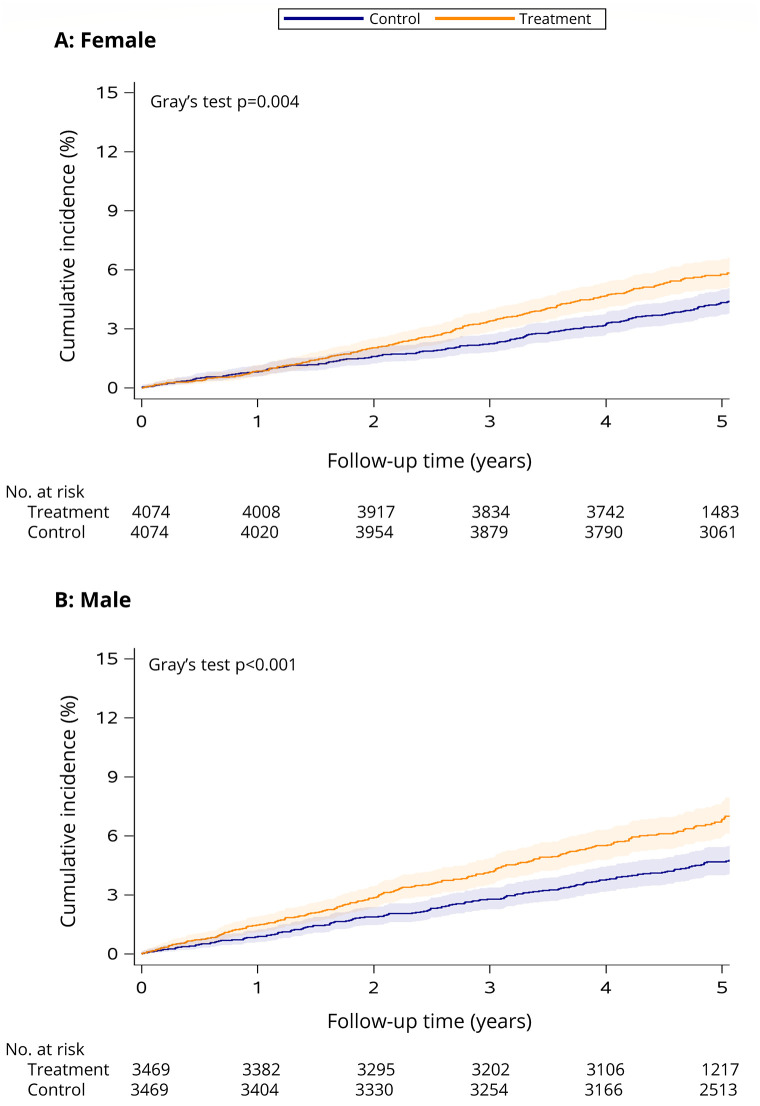
Cumulative incidence of diabetes among female (A) and male (B) smoking cessation treatment patients versus matched controls. The follow-up period begins the day after enrolment in smoking cessation treatment (treatment cohort) or survey completion (control cohort) in 2011/2012 and ends December 31, 2017 (or date of death if it occurred first). Shaded areas indicate the 95% CI. Number of individuals at risk at each time point is presented below the *x* axis.

See Table 1 for the estimated incidence of diabetes within 5 years of follow-up in the treatment and control groups. The cumulative incidence of diabetes was significantly higher over time for treated versus control females (Gray’s test *p* = 0.004, see Fig 3A) and males (Gray’s test *p* < 0.001, see Fig 3B).

### Hypertension

In addition to the common imbalances described above (first paragraph of Results), there were further imbalances between the treatment and control groups in the matched sub-cohort at risk of hypertension. Three prevalent health conditions were more common in the treatment versus control groups: COPD (females: 19.4% vs. 11.6%; males: 17.9% vs. 9.9%), diabetes (females: 7.8% vs. 5.0%; males: 9.2% vs. 5.5%) and asthma (females: 24.7% vs. 19.1%). A lower proportion had 0–5 ADG comorbidities (males: 67.8% vs. 73.7%), and a higher proportion had 10+ ADG comorbidities (males: 7.4% vs. 4.9%), in the treatment group, indicating an overall higher comorbidity burden among treated versus control males. The proportion having at least one outpatient visit in two years prior to index was higher in the treatment group of both sexes (females: 96.0% vs. 92.4%; males: 91.7% vs. 79.0%). Baseline characteristics of the matched sub-cohort at risk of hypertension, stratified by sex, are described in S4 Table.

**Fig 4:**
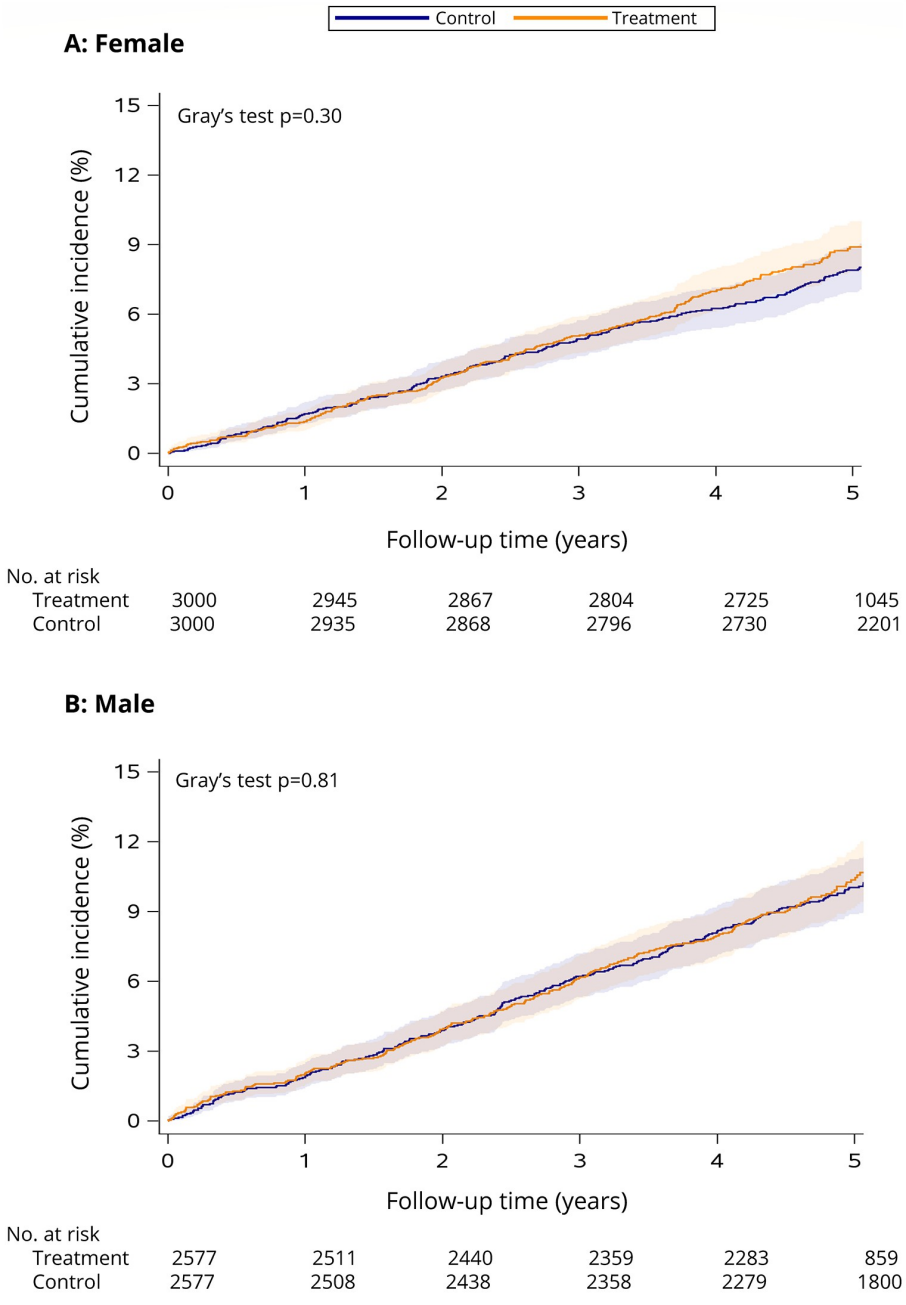
Cumulative incidence of hypertension among female (A) and male (B) smoking cessation treatment patients versus matched controls. The follow-up period begins the day after enrolment in smoking cessation treatment (treatment cohort) or survey completion (control cohort) in 2011/2012 and ends December 31, 2017 (or date of death if it occurred first). Shaded areas indicate the 95% CI. Number of individuals at risk at each time point is presented below the *x* axis.

See Table 1 for the estimated incidence of hypertension within 5 years of follow-up in the treatment and control groups. There was no significant difference in the cumulative incidence of hypertension (females: Gray’s test *p* = 0.30, see Fig 4A; males: Gray’s test *p* = 0.81, see Fig 4B).

### Major cardiovascular events

In addition to the common imbalances described above (first paragraph of Results), there were further imbalances between treatment and control groups in the matched sub-cohort at risk of major cardiovascular events. Three prevalent health conditions were more common in the treatment versus control groups: COPD (females: 30.2% vs. 20.3%; males: 26.2% vs. 17.4%), diabetes (males: 15.7% vs. 10.8%) and asthma (females: 24.0% vs. 18.8%). The proportion having made at least one outpatient visit in two years prior to index was higher among the treated versus control group (females: 96.6% vs. 93.9%; males: 93.8% vs. 83.8%). Baseline characteristics of the matched sub-cohort at risk of major cardiovascular events, stratified by sex, are described in S5 Table.

**Fig 5:**
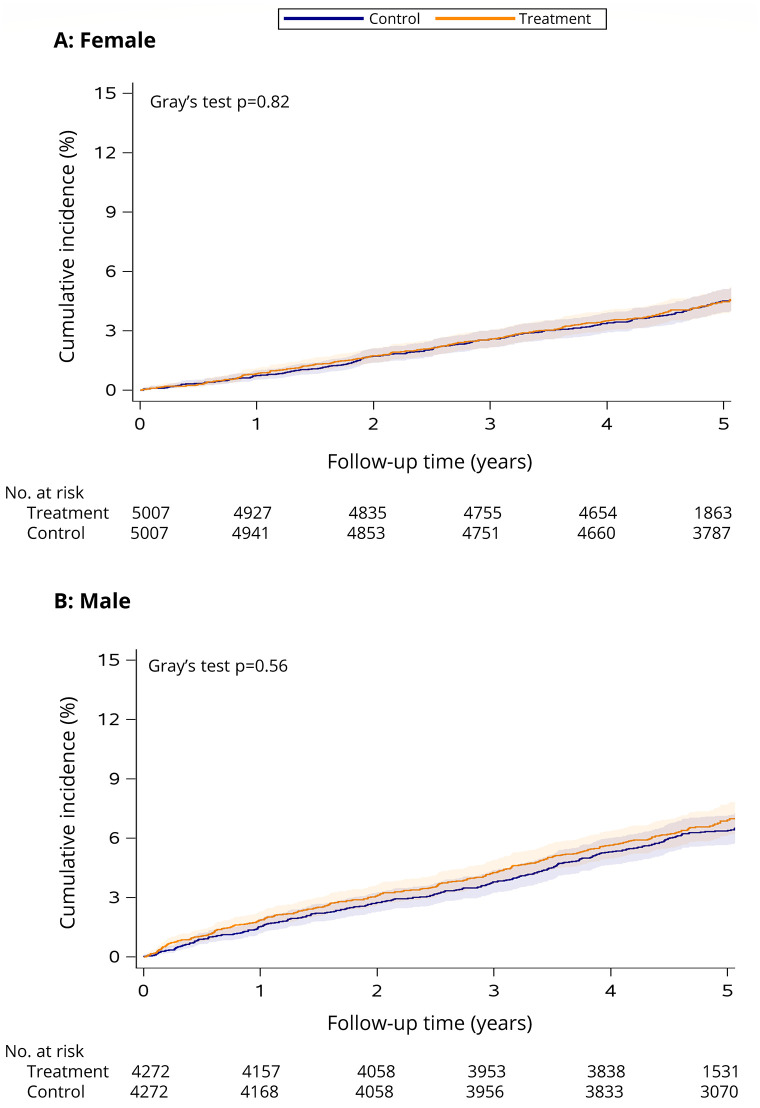
Cumulative incidence of major cardiovascular events among female (A) and male (B) smoking cessation treatment patients versus matched controls. The follow-up period begins the day after enrolment in smoking cessation treatment (treatment cohort) or survey completion (control cohort) in 2011/2012 and ends December 31, 2017 (or date of death if it occurred first). Shaded areas indicate the 95% CI. Number of individuals at risk at each time point is presented below the *x* axis.

See Table 1 for the estimated incidence of major cardiovascular events within 5 years of follow-up in the treatment and control groups. There was no significant difference in the cumulative incidence of major cardiovascular events (females: Gray’s test *p* = 0.82, see Fig 5A; males: Gray’s test *p* = 0.56, see Fig 5B).

## Discussion

In this retrospective matched cohort study, we observed a higher incidence of certain chronic diseases within five years follow-up among Ontarians who smoked and who had enrolled in a smoking cessation treatment program compared to Ontarians who smoke but had not enrolled in the program. Specifically, there was a higher incidence of diabetes among the treatment group versus control group of both sexes, and a higher incidence of COPD among male treatment versus control groups. No difference was observed between treatment and control groups in the incidence of cancer, hypertension, or major cardiovascular events during follow-up.

Increased incidence of diabetes in the group of Ontarians seeking smoking cessation treatment was an expected finding. While cigarette smoking itself is a risk factor for incident diabetes [43], quitting smoking is associated with an increased short-term risk of incident type 2 diabetes compared with continued smoking [44, 45]. This risk was reported to be greatest within 3 years of quitting in one study [44], and between 5 and 7 years after quitting in another study [45], before decreasing over time until there was no excess risk. A reduction in cigarettes smoked per day over 3 years was also associated with increased insulin and glucose levels in males, but not females, in one study [46]. Various analyses suggest this increased risk for diabetes is at least partially explained by weight gain [44–46]. Quit status could not be incorporated into our analyses due to the cross-sectional design of the CCHS and resulting lack of longitudinal smoking status for the control sub-cohorts. However, given the previously reported proportions who quit in the STOP program (27%) [47] versus past-year quit attempt success in the general population (12% in 2017 [48]), the proportion of recent quitters was likely higher in the treatment sub-cohort. This may have driven up the number of incident cases of diabetes in the treatment group relative to the control group. The greater proportion of daily (versus occasional) smoking in the treatment sub-cohort at baseline may also have been associated with a higher incidence of diabetes during follow-up, given the increased risk of diabetes among heavier versus lighter smokers [43]. Regardless of the underlying mechanisms, this finding has important implications for the health care system. It is important that health care providers delivering smoking cessation treatment are aware of an elevated incidence of diabetes in this population and combine smoking cessation interventions with strategies for prevention and early detection of type 2 diabetes [44]. This may include regular monitoring of glucose levels during and following a quit attempt, and recommending treatments associated with less weight gain, particularly for individuals with a higher risk of diabetes. A recent network meta-analysis found several pharmacologic treatments for smoking cessation minimized weight gain, with nicotine patches combined with fluoxetine being associated with the least weight gain [49]. Notably, the increased short-term risk of type 2 diabetes does not negate the beneficial impact of smoking cessation on cardiovascular or all-cause mortality [45].

We also found an increased incidence of COPD in the treatment group compared to the control group, although for males only. At index, treated males had higher recent healthcare utilization and prevalent diabetes compared to control males, whereas treated females appeared to have less comorbidity burden than control females. Greater incidence of COPD in treatment versus control group males, but not females, may have been due to these underlying differences in health factors at index, but differential diagnosis of COPD may also have occurred. A review of several studies, including one conducted in Ontario, suggests that only 20% to 30% of individuals with evidence of persistent airflow limitation on spirometry have been diagnosed with COPD [50], and under diagnosis is more common among males [50]. Thus, cases of COPD that were undiagnosed may have been more common among treatment versus control group males, and as a result, more diagnoses may have occurred in this group during follow-up. The greater proportion of diabetes among treatment versus control group males supports this possibility given that diabetes is more common in patients with COPD [51]; however, shared underlying mechanisms may also put one at risk for both diabetes and COPD [52]. We are unaware of any prior research or mechanism by which smoking cessation treatment, per se, would increase the incidence of COPD, and thus suggest this finding is likely due to extenuating factors. The greater prevalence of COPD at both index (in the other four chronic disease sub-cohorts) and at follow-up in our sample suggest that primary care patients seeking smoking cessation treatment are an at-risk group who should be targeted for COPD case finding. Moreover, a case-finding approach can increase detection of COPD among all patients who smoke, not just those seeking smoking cessation treatment (e.g., see algorithm by Diab et al. [50]). The National Lung Health Education Program Consensus Statement recommends that primary care providers perform spirometry in patients 40 years of age or older who are current or former smokers and have chronic cough, excessive sputum production, wheezing, or shortness of breath out of proportion to age or activity performed [53]. Similar recommendations have been made by others [50].

In contrast to diabetes and COPD, incidence of the three other conditions (cancer, major cardiovascular events, and hypertension) did not differ between the treatment and control groups over follow-up. Smoking cessation reduces risk of cancer, but smoking cessation treatment may not have a similar association. However, incidence of cancer is inversely related to time since cessation [54–58], thus five years may not have been long enough to find a difference in overall cancer incidence following smoking cessation treatment with a 27% cessation rate. Examining overall cancer incidence may have also masked changes in select types of cancer, particularly those most highly linked with smoking. For example, a recent meta-analysis found that a reduction in smoking was associated with lower incidence of lung cancer but did not lower all-cancer or smoking-related cancer risk [8]. Thus, future studies examining the association between smoking cessation treatment and incidence of cancer may wish to examine incidence of select types of cancer as well as all cancers (if feasible to do so). Although smoking cessation is associated with lower risk of cardiovascular disease within five years compared to continued smoking [5], the present study suggests major cardiovascular events are not reduced within five years of smoking cessation treatment. However, COPD, which is associated with higher incidence of both cardiovascular disease and hypertension [51], was higher in the treatment groups relative to the control groups and could have masked any potential reduction in both these disease outcomes following smoking cessation treatment.

Ultimately, imbalances between the treatment and control groups in the matched sub-cohorts prevent us from being able to make firm conclusions regarding the impact of smoking cessation treatment on incident chronic diseases.

Producing real-world evidence is important so that simulation-based modeling exercises are not the only source of population-based information available. However, real-world evidence relies on the availability of real-world data; we were able to leverage circumstances that made such an investigation possible: 1) access to data from a large sample of treatment-seeking people, 2) a large sample of respondents to a population-based survey to serve as controls, 3) both previously linked to routinely collected healthcare administrative data. However, low overall smoking prevalence in the population of Ontario meant that the number of survey respondents who smoked was also relatively low. For this reason, we combined multiple cycles of the CCHS, a common approach used to increase the number of potential controls. STOP, being a treatment program, tends to serve those who smoke more heavily overall (and relatively few of those who smoke lightly or non-daily), but the CCHS, being a population-based survey, has a broader distribution of smoking behaviors. To achieve a more appropriate comparison, we 1:1 matched on age and sex, and propensity score-matched with a caliper width of 0.2 standard deviations of the estimated propensity score logit. As described above, imbalances remained on some smoking measures and some prevalent comorbidities. Decreasing the caliper width would have resulted in pairs that were more tightly matched but with a resulting decrease in the number of matched pairs (i.e., greater internal validity but decreased external validity). As is, we obtained matches for 76.8% of the STOP participants whose data was linked. Thus, we chose an approach that balanced internal and external validity.

The current study highlights the challenge in using existing real-world data to obtain a control group of people who smoke with which to compare those who smoke and seek treatment. To use population-based surveys, as we have done, those surveys would need to be extraordinarily large and, likely, would need to oversample those who smoke more heavily and/or who have made quit attempts. Alternatively, context-specific surveys of people who smoke could be used if the survey data was then linked to existing healthcare administrative data. Linkage of survey data from large numbers of people has obvious cost implications and cross-sectional data capture only a snapshot in time. But using survey data to identify controls is only necessary because smoking status, though central to obtaining a fulsome patient history, isn’t easily obtained from existing healthcare datasets. Indeed, many important patient characteristics, like smoking behaviours, are captured only in free text fields as case notes in special sections of electronic medical records; the unstructured nature of these data present an obstacle for use in producing real world evidence [59]. Annually, at least 80% of Ontarians access the publicly funded healthcare system and as a result an administrative record is generated [60]. If smoking behaviours and history (e.g., smoking status, cigarettes/day, age first smoked), were routinely captured in a usable format, we would have had over 2 million (roughly 19% of Ontario’s population in 2011/2012 [61, 62]) Ontarians as potential controls. Further, and though outside the scope of the current study, other traditional health behaviours that are difficult to measure using existing administrative data (i.e., alcohol consumption, poor diet, physical inactivity, lack of sleep) are also poorly ascertained yet important determinants of health and healthcare service use. Improving capture of these types of factors would be a significant leap forward in the ability to use real world data to produce real world evidence.

As discussed above, the main limitation of the current study is that individuals were not randomized to groups and despite matching, the treatment and control group in the matched sub-cohorts were not balanced on several baseline variables. Most importantly, there was imbalance on conditions known to be associated with disease outcomes. On the other hand, a strength of the current study was that data from the treatment group was collected from patients receiving smoking cessation treatment under real-world circumstances with their primary care provider, enhancing the generalizability of our findings to real-world treatment in healthcare settings. Further, as some individuals in the control group likely sought treatment (e.g., over the counter NRT or other prescription medications such as varenicline), the current study is not a comparison of any versus no smoking cessation treatment, but speaks more specifically to the outcomes associated with delivering a smoking cessation treatment program providing NRT with behavioural support in primary care, in a context where other treatments are available. Lastly, our study speaks to incident chronic disease within a five-year follow-up period and cannot rule out the possibility that differences presented after that timeframe. Therefore, future studies should extend the observation period.

## Conclusions

In summary, further research able to adequately control for baseline differences between those who do and do not seek smoking cessation treatment is needed using real-world data to establish whether there is a differential change in incidence of chronic disease in the short and long term. Although there was imbalance between the treatment and control groups, our study shows those seeking smoking cessation treatment have higher levels of comorbidity than population-based controls, both prior to and following treatment. Health care providers should be aware of higher incidence of diabetes and COPD among those seeking smoking cessation treatment and conduct appropriate screening for these conditions.

## Supporting information

S1 Checklist RECORD checklist. (DOCX)

S1 Fig Derivation of at-risk matched treatment and control cohorts. (DOCX)

S1 Table Baseline characteristics of matched treatment and control females and males, at risk for cancer. (DOCX)

S2 Table Baseline characteristics of matched treatment and control females and males, at risk for chronic obstructive pulmonary disease. (DOCX)

S3 Table Baseline characteristics of matched treatment and control females and males, at risk for diabetes. (DOCX)

S4 Table Baseline characteristics of matched treatment and control females and males, at risk for hypertension. (DOCX)

S5 Table Baseline characteristics of matched treatment and control females and males, at risk for major cardiovascular events. (DOCX)
