# Supplementary material for: Incidence of chronic disease following smoking cessation treatment: A matched cohort study using linked administrative healthcare data in Ontario, Canada
Source: PLoS One. 2023 Jul 26;18(7):e0288759. doi: 10.1371/journal.pone.0288759 (PMC10370896; doi:10.1371/journal.pone.0288759)
Supplement: S1 Fig — (DOCX) [file pone.0288759.s002.docx]

**S1 Figure.** Derivation of matched treatment and control at-risk cohorts

**Control Cohort (CCHS)**

**Treatment Cohort (STOP)**

▪ CCHS respondents in 2011/12,

2009/10 and 2007/08 cycles

▪ Consented to linkage and could be

linked

**N=100,565**

▪ STOP patients who enrolled in FHTs,

CHCs, and AAs between 01Jul2011 to

31Dec2012

▪ Consented to linkage (90% of original

sample) and whose data could be

linked (96% linkage rate)

▪ First enrolment during this timeframe,

excluding subsequent enrolments

**N=13,561**

▪ Limit to respondents that smoked at

time of the survey

**N=19,771**

▪ Limit to patients that smoked daily or

occasionally at enrolment

**N=13,318**

**Excluded (n=511):**

▪ Invalid record or sex missing, age <12

or >105 years, death before index date,

or in STOP treatment cohort (179)

▪ Non-Ontario residents or not eligible for

OHIP at index date (86)

▪ Without full 2 year lookback (246)

**Excluded (n=353):**

▪ Invalid record or sex missing, age <12

or >105 years, death before index date,

or in CCHS control cohort with non-

smoker status (24)

▪ Non-Ontario residents or not eligible for

OHIP at index date (127)

▪ Without full 2 year lookback (202)

▪ Potential control cohort available for

matching

**N=19,260**

▪ Potential treatment cohort available for

matching

**N=12,965**

▪ **Matched control cohort**

**N=9,951** (5,257 females; 4,694 males)

▪ **Matched treatment cohort**

**N=9,951** (5,257 females; 4,694 males)

**Matched at-risk cohorts**

Treatment–control pairs at risk for chronic disease outcome:

**Major CV events**

**N=9,279 pairs**

(5,007 female;

4,272 male)

**Hypertension**

**N=5,577 pairs**

(3,000 female; 2,577 male)

**Diabetes**

**N=7,543 pairs**

(4,074 female; 3,469 male)

**Cancer**

**N=9,134 pairs**

(4,832 female; 4,302 male)

**COPD**

**N=5,905 pairs**

(3,024 female; 2,881 male)

**Abbreviations:** AA = Addiction Agency; CCHS = Canadian Community Health Survey; CHC = Community Health Centre; CV = cardiovascular; FHT = Family Health Team; OHIP = Ontario Health Insurance Plan; STOP = Smoking Treatment for Ontario Patients program.
