# Supplementary material for: Incidence of chronic disease following smoking cessation treatment: A matched cohort study using linked administrative healthcare data in Ontario, Canada
Source: PLoS One. 2023 Jul 26;18(7):e0288759. doi: 10.1371/journal.pone.0288759 (PMC10370896; doi:10.1371/journal.pone.0288759)
Supplement: S1 Table — (DOCX) [file pone.0288759.s003.docx]

**S1 Table.** Baseline characteristics of matched treatment and control females and males, at risk for cancer at index date

|  | **Female** | | | **Male** | | |
| --- | --- | --- | --- | --- | --- | --- |
|  | Treatment cohort  (n=4,832) | Control  cohort  (n=4,832) | SMD | Treatment cohort  (n=4,302) | Control  cohort  (n=4,302) | SMD |
| **Sociodemographic characteristics** |  |  |  |  |  |  |
| Age, mean ± SD | 47.83 ± 14.23 | 47.86 ± 14.24 | 0 | 48.07 ± 13.68 | 48.09 ± 13.66 | 0 |
| Education quintile |  |  |  |  |  |  |
| Missing | 253 (5.2) | 259 (5.4) | 0.01 | 260 (6.0) | 247 (5.7) | 0.01 |
| Q1 (lowest) | 321 (6.6) | 333 (6.9) | 0.01 | 318 (7.4) | 278 (6.5) | 0.04 |
| Q2 | 743 (15.4) | 712 (14.7) | 0.02 | 640 (14.9) | 629 (14.6) | 0.01 |
| Q3 | 947 (19.6) | 958 (19.8) | 0.01 | 881 (20.5) | 854 (19.9) | 0.02 |
| Q4 | 1,226 (25.4) | 1,216 (25.2) | 0 | 1,071 (24.9) | 1,109 (25.8) | 0.02 |
| Q5 (highest) | 1,342 (27.8) | 1,354 (28.0) | 0.01 | 1,132 (26.3) | 1,185 (27.5) | 0.03 |
| Employment quintile |  |  |  |  |  |  |
| Missing | 253 (5.2) | 259 (5.4) | 0.01 | 260 (6.0) | 247 (5.7) | 0.01 |
| Q1 (lowest) | 1,080 (22.4) | 1,078 (22.3) | 0 | 948 (22.0) | 971 (22.6) | 0.01 |
| Q2 | 983 (20.3) | 910 (18.8) | 0.04 | 866 (20.1) | 813 (18.9) | 0.03 |
| Q3 | 827 (17.1) | 932 (19.3) | 0.06 | 809 (18.8) | 833 (19.4) | 0.01 |
| Q4 | 869 (18.0) | 869 (18.0) | 0 | 735 (17.1) | 716 (16.6) | 0.01 |
| Q5 (highest) | 820 (17.0) | 784 (16.2) | 0.02 | 684 (15.9) | 722 (16.8) | 0.02 |
| Rurality + neighbourhood income quintile |  |  |  |  |  |  |
| Missing | 11 (0.2) | 13 (0.3) | 0.01 | ≤ 5 (0.1) | 6 (0.1) | 0.01 |
| Rural | 1,111 (23.0) | 1,123 (23.2) | 0.01 | 1,054 (24.5) | 1,062 (24.7) | 0 |
| Urban Q1 (lowest) | 1,072 (22.2) | 1,070 (22.1) | 0 | 959 (22.3) | 939 (21.8) | 0.01 |
| Urban Q2 | 827 (17.1) | 817 (16.9) | 0.01 | 627 (14.6) | 651 (15.1) | 0.02 |
| Urban Q3 | 693 (14.3) | 725 (15.0) | 0.02 | 625 (14.5) | 633 (14.7) | 0.01 |
| Urban Q4 | 656 (13.6) | 616 (12.7) | 0.02 | 581 (13.5) | 581 (13.5) | 0 |
| Urban Q5 (highest) | 462 (9.6) | 468 (9.7) | 0 | 451 (10.5) | 430 (10.0) | 0.02 |
| Migrant status |  |  |  |  |  |  |
| Immigrant^a^ | 99 (2.0) | 99 (2.0) | 0 | 150 (3.5) | 152 (3.5) | 0 |
| Non-immigrant | 4,733 (98.0) | 4,733 (98.0) | 0 | 4,152 (96.5) | 4,150 (96.5) | 0 |
| **Smoking characteristics** |  |  |  |  |  |  |
| Frequency of smoking |  |  |  |  |  |  |
| Daily | **4,739 (98.1)** | **4,406 (91.2)** | **0.31** | **4,210 (97.9)** | **3,973 (92.4)** | **0.26** |
| Occasional | **93 (1.9)** | **426 (8.8)** | **0.31** | **92 (2.1)** | **329 (7.6)** | **0.26** |
| Cigarettes per day, mean ± SD | 16.38 ± 8.97 | 15.72 ± 8.65 | 0.07 | 19.44 ± 10.28 | 19.58 ± 10.17 | 0.01 |
| Age first tried smoking, mean ± SD | 15.94 ± 4.84 | 15.96 ± 4.80 | 0 | 15.66 ± 4.81 | 15.36 ± 4.44 | 0.07 |
| Duration smoking (years), mean ± SD | 31.89 ± 14.03 | 31.90 ± 13.77 | 0 | 32.41 ± 14.42 | 32.73 ± 14.19 | 0.02 |
| **Health comorbidities** |  |  |  |  |  |  |
| Prevalent comorbidities |  |  |  |  |  |  |
| COPD | **1,425 (29.5)** | **955 (19.8)** | **0.23** | **1,140 (26.5)** | **743 (17.3)** | **0.22** |
| Hypertension | 1,249 (25.8) | 1,238 (25.6) | 0.01 | 1,237 (28.8) | 1,064 (24.7) | 0.09 |
| Diabetes | 624 (12.9) | 500 (10.3) | 0.08 | **697 (16.2)** | **489 (11.4)** | **0.14** |
| Asthma | **1,174 (24.3)** | **916 (19.0)** | **0.13** | 573 (13.3) | 446 (10.4) | 0.09 |
| Cancer | 0 | 0 | 0 | 0 | 0 | 0 |
| Myocardial infarction | 83 (1.7) | 52 (1.1) | 0.05 | 202 (4.7) | 147 (3.4) | 0.06 |
| Congestive heart failure | 97 (2.0) | 81 (1.7) | 0.02 | 101 (2.3) | 74 (1.7) | 0.04 |
| No. ADG comorbidities, mean ± SD |  |  |  |  |  |  |
| 0-5 | 2,232 (46.2) | 2,077 (43.0) | 0.06 | 2,640 (61.4) | 2,811 (65.3) | 0.08 |
| 6-9 | 1,787 (37.0) | 1,903 (39.4) | 0.05 | 1,220 (28.4) | 1,116 (25.9) | 0.05 |
| 10+ | 813 (16.8) | 852 (17.6) | 0.02 | 442 (10.3) | 375 (8.7) | 0.05 |
| **Healthcare service use^b^** |  |  |  |  |  |  |
| Outpatient visits |  |  |  |  |  |  |
| Any outpatient visit | **4,666 (96.6)** | **4,529 (93.7)** | **0.13** | **4,040 (93.9)** | **3,600 (83.7)** | **0.33** |
| Mean ± SD rate ppy | 6.97 ± 8.55 | 6.95 ± 7.78 | 0 | **5.74 ± 8.44** | **4.84 ± 7.28** | **0.11** |
| ED visits |  |  |  |  |  |  |
| Any ED visit | 2,890 (59.8) | 2,693 (55.7) | 0.08 | **2,524 (58.7)** | **2,183 (50.7)** | **0.16** |
| Mean ± SD rate ppy | 0.94 ± 1.49 | 0.91 ± 1.56 | 0.02 | 0.82 ± 1.28 | 0.74 ± 1.39 | 0.06 |
| Hospitalizations |  |  |  |  |  |  |
| Any hospitalization | 795 (16.5) | 839 (17.4) | 0.02 | 641 (14.9) | 502 (11.7) | 0.1 |
| Mean ± SD rate ppy | 0.12 ± 0.36 | 0.13 ± 0.37 | 0.02 | 0.12 ± 0.36 | 0.10 ± 0.33 | 0.06 |

Note. Number (%) are reported unless otherwise noted. **Bolded SMD values are > 0.1 and indicate imbalance between cohorts.** Abbreviations: ADG = Aggregated Diagnostic Groups; COPD = chronic obstructive pulmonary disease; SD = standard deviation; ppy = per person year; ED = emergency department; Q = quintile; IQR = interquartile range; SMD = standardized mean difference.

^a^ Includes immigrants and refugees.

^b^ During 2 year period up to index date.
