# Supplementary material for: Incidence of chronic disease following smoking cessation treatment: A matched cohort study using linked administrative healthcare data in Ontario, Canada
Source: PLoS One. 2023 Jul 26;18(7):e0288759. doi: 10.1371/journal.pone.0288759 (PMC10370896; doi:10.1371/journal.pone.0288759)
Supplement: S2 Table — (DOCX) [file pone.0288759.s004.docx]

**S2 Table.** Baseline characteristics of matched treatment and control females and males, at risk for chronic obstructive pulmonary disease at index date

|  | **Female** | | | **Male** | | |
| --- | --- | --- | --- | --- | --- | --- |
|  | Treatment cohort  (n=3,024) | Control  cohort  (n=3,024) | SMD | Treatment cohort  (n=2,881) | Control  cohort  (n=2,881) | SMD |
| **Sociodemographic characteristics** |  |  |  |  |  |  |
| Age, mean ± SD | 41.69 ± 13.15 | 41.71 ± 13.16 | 0 | 43.51 ± 13.23 | 43.55 ± 13.19 | 0 |
| Education quintile |  |  |  |  |  |  |
| Missing | 173 (5.7) | 176 (5.8) | 0 | 197 (6.8) | 174 (6.0) | 0.03 |
| Q1 (lowest) | 211 (7.0) | 200 (6.6) | 0.01 | 229 (7.9) | 193 (6.7) | 0.05 |
| Q2 | 440 (14.6) | 445 (14.7) | 0 | 448 (15.6) | 429 (14.9) | 0.02 |
| Q3 | 592 (19.6) | 577 (19.1) | 0.01 | 558 (19.4) | 563 (19.5) | 0 |
| Q4 | 763 (25.2) | 766 (25.3) | 0 | 704 (24.4) | 726 (25.2) | 0.02 |
| Q5 (highest) | 845 (27.9) | 860 (28.4) | 0.01 | 745 (25.9) | 796 (27.6) | 0.04 |
| Employment quintile |  |  |  |  |  |  |
| Missing | 173 (5.7) | 176 (5.8) | 0 | 197 (6.8) | 174 (6.0) | 0.03 |
| Q1 (lowest) | 665 (22.0) | 671 (22.2) | 0 | 632 (21.9) | 640 (22.2) | 0.01 |
| Q2 | 620 (20.5) | 555 (18.4) | 0.05 | 546 (19.0) | 512 (17.8) | 0.03 |
| Q3 | 505 (16.7) | 572 (18.9) | 0.06 | 551 (19.1) | 570 (19.8) | 0.02 |
| Q4 | 536 (17.7) | 572 (18.9) | 0.03 | 485 (16.8) | 514 (17.8) | 0.03 |
| Q5 (highest) | 525 (17.4) | 478 (15.8) | 0.04 | 470 (16.3) | 471 (16.3) | 0 |
| Rurality + neighbourhood income quintile |  |  |  |  |  |  |
| Missing | 6 (0.2) | ≤ 5 (0.2) | 0.01 | 6 (0.2) | ≤ 5 (0.1) | 0.03 |
| Rural | 660 (21.8) | 692 (22.9) | 0.03 | 676 (23.5) | 702 (24.4) | 0.02 |
| Urban Q1 (lowest) | 654 (21.6) | 659 (21.8) | 0 | 625 (21.7) | 606 (21.0) | 0.02 |
| Urban Q2 | 535 (17.7) | 495 (16.4) | 0.04 | 413 (14.3) | 435 (15.1) | 0.02 |
| Urban Q3 | 452 (14.9) | 485 (16.0) | 0.03 | 444 (15.4) | 438 (15.2) | 0.01 |
| Urban Q4 | 419 (13.9) | 394 (13.0) | 0.02 | 400 (13.9) | 397 (13.8) | 0 |
| Urban Q5 (highest) | 298 (9.9) | 294 (9.7) | 0 | 317 (11.0) | 300 (10.4) | 0.02 |
| Migrant status |  |  |  |  |  |  |
| Immigrant^a^ | 79 (2.6) | 67 (2.2) | 0.03 | 121 (4.2) | 123 (4.3) | 0 |
| Non-immigrant | 2,945 (97.4) | 2,957 (97.8) | 0.03 | 2,760 (95.8) | 2,758 (95.7) | 0 |
| **Smoking characteristics** |  |  |  |  |  |  |
| Frequency of smoking |  |  |  |  |  |  |
| Daily | **2,963 (98.0)** | **2,706 (89.5)** | **0.36** | **2,824 (98.0)** | **2,646 (91.8)** | **0.28** |
| Occasional | **61 (2.0)** | **318 (10.5)** | **0.36** | **57 (2.0)** | **235 (8.2)** | **0.28** |
| Cigarettes per day, mean ± SD | **15.48 ± 8.19** | **14.42 ± 7.88** | **0.13** | 18.86 ± 9.73 | 18.90 ± 9.98 | 0 |
| Age first tried smoking, mean ± SD | 15.56 ± 4.16 | 15.51 ± 4.04 | 0.01 | 15.85 ± 4.71 | 15.40 ± 4.38 | 0.1 |
| Duration smoking (years), mean ± SD | 26.13 ± 13.00 | 26.20 ± 12.77 | 0.01 | 27.66 ± 13.70 | 28.14 ± 13.54 | 0.04 |
| **Health comorbidities** |  |  |  |  |  |  |
| Prevalent comorbidities |  |  |  |  |  |  |
| COPD | 0 | 0 | 0 | 0 | 0 | 0 |
| Hypertension | 470 (15.5) | 488 (16.1) | 0.02 | 591 (20.5) | 519 (18.0) | 0.06 |
| Diabetes | 276 (9.1) | 201 (6.6) | 0.09 | **362 (12.6)** | **243 (8.4)** | **0.14** |
| Asthma | 620 (20.5) | 530 (17.5) | 0.08 | 351 (12.2) | 295 (10.2) | 0.06 |
| Cancer | 61 (2.0) | 73 (2.4) | 0.03 | 77 (2.7) | 59 (2.0) | 0.04 |
| Myocardial infarction | 31 (1.0) | 20 (0.7) | 0.04 | 94 (3.3) | 55 (1.9) | 0.09 |
| Congestive heart failure | 15 (0.5) | 11 (0.4) | 0.02 | 23 (0.8) | 20 (0.7) | 0.01 |
| No. ADG comorbidities, mean ± SD |  |  |  |  |  |  |
| 0-5 | **1,548 (51.2)** | **1,352 (44.7)** | **0.13** | 1,979 (68.7) | 2,052 (71.2) | 0.06 |
| 6-9 | 1,090 (36.0) | 1,211 (40.0) | 0.08 | 686 (23.8) | 640 (22.2) | 0.04 |
| 10+ | 386 (12.8) | 461 (15.2) | 0.07 | 216 (7.5) | 189 (6.6) | 0.04 |
| **Healthcare service use^b^** |  |  |  |  |  |  |
| Outpatient visits |  |  |  |  |  |  |
| Any outpatient visit | 2,895 (95.7) | 2,829 (93.6) | 0.1 | **2,654 (92.1)** | **2,327 (80.8)** | **0.34** |
| Mean ± SD rate ppy | 6.65 ± 9.08 | 6.63 ± 7.93 | 0 | **5.22 ± 8.83** | **4.27 ± 7.39** | **0.12** |
| ED visits |  |  |  |  |  |  |
| Any ED visit | 1,796 (59.4) | 1,735 (57.4) | 0.04 | **1,667 (57.9)** | **1,459 (50.6)** | **0.15** |
| Mean ± SD rate ppy | 0.93 ± 1.50 | 0.95 ± 1.56 | 0.02 | 0.79 ± 1.26 | 0.73 ± 1.39 | 0.05 |
| Hospitalizations |  |  |  |  |  |  |
| Any hospitalization | 467 (15.4) | 539 (17.8) | 0.06 | 334 (11.6) | 276 (9.6) | 0.07 |
| Mean ± SD rate ppy | 0.11 ± 0.31 | 0.12 ± 0.33 | 0.05 | 0.08 ± 0.28 | 0.07 ± 0.26 | 0.05 |

Note. Number (%) are reported unless otherwise noted. **Bolded SMD values are > 0.1 and indicate imbalance between cohorts.** Abbreviations: ADG = Aggregated Diagnostic Groups; COPD = chronic obstructive pulmonary disease; SD = standard deviation; ppy = per person year; ED = emergency department; Q = quintile; IQR = interquartile range; SMD = standardized mean difference.

^a^ Includes immigrants and refugees.

^b^ During 2 year period up to index date.
