# Supplementary material for: Incidence of chronic disease following smoking cessation treatment: A matched cohort study using linked administrative healthcare data in Ontario, Canada
Source: PLoS One. 2023 Jul 26;18(7):e0288759. doi: 10.1371/journal.pone.0288759 (PMC10370896; doi:10.1371/journal.pone.0288759)
Supplement: S3 Table — (DOCX) [file pone.0288759.s005.docx]

**S3 Table.** Baseline characteristics of matched treatment and control females and males, at risk for diabetes at index date

|  | **Female** | | | **Male** | | |
| --- | --- | --- | --- | --- | --- | --- |
|  | Treatment cohort  (n=4,074) | Control  cohort  (n=4,074) | SMD | Treatment cohort  (n=3,469) | Control  cohort  (n=3,469) | SMD |
| **Sociodemographic characteristics** |  |  |  |  |  |  |
| Age, mean ± SD | 46.47 ± 14.21 | 46.50 ± 14.22 | 0 | 46.36 ± 13.80 | 46.40 ± 13.78 | 0 |
| Education quintile |  |  |  |  |  |  |
| Missing | 221 (5.4) | 219 (5.4) | 0 | 214 (6.2) | 202 (5.8) | 0.01 |
| Q1 (lowest) | 263 (6.5) | 273 (6.7) | 0.01 | 270 (7.8) | 223 (6.4) | 0.05 |
| Q2 | 623 (15.3) | 601 (14.8) | 0.02 | 515 (14.8) | 518 (14.9) | 0 |
| Q3 | 781 (19.2) | 801 (19.7) | 0.01 | 680 (19.6) | 690 (19.9) | 0.01 |
| Q4 | 1,034 (25.4) | 1,018 (25.0) | 0.01 | 884 (25.5) | 879 (25.3) | 0 |
| Q5 (highest) | 1,152 (28.3) | 1,162 (28.5) | 0.01 | 906 (26.1) | 957 (27.6) | 0.03 |
| Employment quintile |  |  |  |  |  |  |
| Missing | 221 (5.4) | 219 (5.4) | 0 | 214 (6.2) | 202 (5.8) | 0.01 |
| Q1 (lowest) | 907 (22.3) | 908 (22.3) | 0 | 751 (21.6) | 761 (21.9) | 0.01 |
| Q2 | 826 (20.3) | 772 (18.9) | 0.03 | 694 (20.0) | 641 (18.5) | 0.04 |
| Q3 | 681 (16.7) | 779 (19.1) | 0.06 | 636 (18.3) | 663 (19.1) | 0.02 |
| Q4 | 739 (18.1) | 731 (17.9) | 0.01 | 600 (17.3) | 615 (17.7) | 0.01 |
| Q5 (highest) | 700 (17.2) | 665 (16.3) | 0.02 | 574 (16.5) | 587 (16.9) | 0.01 |
| Rurality + neighbourhood income quintile |  |  |  |  |  |  |
| Missing | 7 (0.2) | 10 (0.2) | 0.02 | ≤ 5 (0.1) | ≤ 5 (0.1) | 0 |
| Rural | 949 (23.3) | 938 (23.0) | 0.01 | 859 (24.8) | 864 (24.9) | 0 |
| Urban Q1 (lowest) | 872 (21.4) | 862 (21.2) | 0.01 | 767 (22.1) | 705 (20.3) | 0.04 |
| Urban Q2 | 698 (17.1) | 691 (17.0) | 0 | 494 (14.2) | 529 (15.2) | 0.03 |
| Urban Q3 | 594 (14.6) | 637 (15.6) | 0.03 | 501 (14.4) | 533 (15.4) | 0.03 |
| Urban Q4 | 546 (13.4) | 536 (13.2) | 0.01 | 465 (13.4) | 480 (13.8) | 0.01 |
| Urban Q5 (highest) | 408 (10.0) | 400 (9.8) | 0.01 | 378 (10.9) | 353 (10.2) | 0.02 |
| Migrant status |  |  |  |  |  |  |
| Immigrant^a^ | 90 (2.2) | 90 (2.2) | 0 | 115 (3.3) | 129 (3.7) | 0.02 |
| Non-immigrant | 3,984 (97.8) | 3,984 (97.8) | 0 | 3,354 (96.7) | 3,340 (96.3) | 0.02 |
| **Smoking characteristics** |  |  |  |  |  |  |
| Frequency of smoking |  |  |  |  |  |  |
| Daily | **3,992 (98.0)** | **3,698 (90.8)** | **0.32** | **3,396 (97.9)** | **3,199 (92.2)** | **0.26** |
| Occasional | **82 (2.0)** | **376 (9.2)** | **0.32** | **73 (2.1)** | **270 (7.8)** | **0.26** |
| Cigarettes per day, mean ± SD | 15.81 ± 8.38 | 15.29 ± 8.43 | 0.06 | 19.10 ± 9.90 | 19.34 ± 9.90 | 0.02 |
| Age first tried smoking, mean ± SD | 15.83 ± 4.64 | 15.87 ± 4.59 | 0.01 | 15.67 ± 4.66 | 15.37 ± 4.29 | 0.07 |
| Duration smoking (years), mean ± SD | 30.64 ± 14.02 | 30.64 ± 13.79 | 0 | 30.69 ± 14.48 | 31.03 ± 14.18 | 0.02 |
| **Health comorbidities** |  |  |  |  |  |  |
| Prevalent comorbidities |  |  |  |  |  |  |
| COPD | **1,111 (27.3)** | **716 (17.6)** | **0.23** | **851 (24.5)** | **522 (15.0)** | **0.24** |
| Hypertension | 870 (21.4) | 884 (21.7) | 0.01 | 809 (23.3) | 719 (20.7) | 0.06 |
| Diabetes | 0 | 0 | 0 | 0 | 0 | 0 |
| Asthma | **950 (23.3)** | **733 (18.0)** | **0.13** | 472 (13.6) | 360 (10.4) | 0.1 |
| Cancer | 140 (3.4) | 140 (3.4) | 0 | 134 (3.9) | 109 (3.1) | 0.04 |
| Myocardial infarction | 59 (1.4) | 35 (0.9) | 0.06 | 114 (3.3) | 77 (2.2) | 0.07 |
| Congestive heart failure | 53 (1.3) | 45 (1.1) | 0.02 | 56 (1.6) | 37 (1.1) | 0.05 |
| No. ADG comorbidities, mean ± SD |  |  |  |  |  |  |
| 0-5 | 1,932 (47.4) | 1,807 (44.4) | 0.06 | 2,192 (63.2) | 2,340 (67.5) | 0.09 |
| 6-9 | 1,500 (36.8) | 1,591 (39.1) | 0.05 | 941 (27.1) | 876 (25.3) | 0.04 |
| 10+ | 642 (15.8) | 676 (16.6) | 0.02 | 336 (9.7) | 253 (7.3) | 0.09 |
| **Healthcare service use^b^** |  |  |  |  |  |  |
| Outpatient visits |  |  |  |  |  |  |
| Any outpatient visit | **3,923 (96.3)** | **3,809 (93.5)** | **0.13** | **3,233 (93.2)** | **2,847 (82.1)** | **0.34** |
| Mean ± SD rate ppy | 6.72 ± 8.39 | 6.68 ± 7.78 | 0.01 | **5.46 ± 8.76** | **4.46 ± 7.37** | **0.12** |
| ED visits |  |  |  |  |  |  |
| Any ED visit | 2,445 (60.0) | 2,241 (55.0) | 0.1 | **2,052 (59.2)** | **1,749 (50.4)** | **0.18** |
| Mean ± SD rate ppy | 0.91 ± 1.44 | 0.87 ± 1.45 | 0.03 | 0.83 ± 1.28 | 0.74 ± 1.45 | 0.06 |
| Hospitalizations |  |  |  |  |  |  |
| Any hospitalization | 656 (16.1) | 704 (17.3) | 0.03 | **468 (13.5)** | **349 (10.1)** | **0.11** |
| Mean ± SD rate ppy | 0.12 ± 0.34 | 0.13 ± 0.35 | 0.02 | 0.11 ± 0.34 | 0.08 ± 0.29 | 0.09 |

Note. Number (%) are reported unless otherwise noted. **Bolded SMD values are > 0.1 and indicate imbalance between cohorts.** Abbreviations: ADG = Aggregated Diagnostic Groups; COPD = chronic obstructive pulmonary disease; SD = standard deviation; ppy = per person year; ED = emergency department; Q = quintile; IQR = interquartile range; SMD = standardized mean difference.

^a^ Includes immigrants and refugees.

^b^ During 2 year period up to index date.
