# Supplementary material for: Incidence of chronic disease following smoking cessation treatment: A matched cohort study using linked administrative healthcare data in Ontario, Canada
Source: PLoS One. 2023 Jul 26;18(7):e0288759. doi: 10.1371/journal.pone.0288759 (PMC10370896; doi:10.1371/journal.pone.0288759)
Supplement: S4 Table — (DOCX) [file pone.0288759.s006.docx]

**S4 Table.** Baseline characteristics of matched treatment and control females and males, at risk for hypertension at index date

|  | **Female** | | | **Male** | | |
| --- | --- | --- | --- | --- | --- | --- |
|  | Treatment cohort  (n=3,000) | Control  cohort  (n=3,000) | SMD | Treatment cohort  (n=2,577) | Control  cohort  (n=2,577) | SMD |
| **Sociodemographic characteristics** |  |  |  |  |  |  |
| Age, mean ± SD | 41.80 ± 12.72 | 41.82 ± 12.73 | 0 | 42.31 ± 12.68 | 42.35 ± 12.66 | 0 |
| Education quintile |  |  |  |  |  |  |
| Missing | 164 (5.5) | 168 (5.6) | 0.01 | 153 (5.9) | 143 (5.5) | 0.02 |
| Q1 (lowest) | 198 (6.6) | 195 (6.5) | 0 | 222 (8.6) | 167 (6.5) | 0.08 |
| Q2 | 456 (15.2) | 423 (14.1) | 0.03 | 375 (14.6) | 390 (15.1) | 0.02 |
| Q3 | 572 (19.1) | 592 (19.7) | 0.02 | 534 (20.7) | 520 (20.2) | 0.01 |
| Q4 | 786 (26.2) | 760 (25.3) | 0.02 | 641 (24.9) | 652 (25.3) | 0.01 |
| Q5 (highest) | 824 (27.5) | 862 (28.7) | 0.03 | 652 (25.3) | 705 (27.4) | 0.05 |
| Employment quintile |  |  |  |  |  |  |
| Missing | 164 (5.5) | 168 (5.6) | 0.01 | 153 (5.9) | 143 (5.5) | 0.02 |
| Q1 (lowest) | 669 (22.3) | 671 (22.4) | 0 | 568 (22.0) | 575 (22.3) | 0.01 |
| Q2 | 633 (21.1) | 541 (18.0) | 0.08 | 517 (20.1) | 488 (18.9) | 0.03 |
| Q3 | 497 (16.6) | 585 (19.5) | 0.08 | 501 (19.4) | 491 (19.1) | 0.01 |
| Q4 | 535 (17.8) | 569 (19.0) | 0.03 | 433 (16.8) | 453 (17.6) | 0.02 |
| Q5 (highest) | 502 (16.7) | 466 (15.5) | 0.03 | 405 (15.7) | 427 (16.6) | 0.02 |
| Rurality + neighbourhood income quintile |  |  |  |  |  |  |
| Missing | 7 (0.2) | 10 (0.3) | 0.02 | ≤ 5 (0.2) | ≤ 5 (0.1) | 0.02 |
| Rural | 670 (22.3) | 684 (22.8) | 0.01 | 584 (22.7) | 670 (26.0) | 0.08 |
| Urban Q1 (lowest) | 667 (22.2) | 669 (22.3) | 0 | 580 (22.5) | 548 (21.3) | 0.03 |
| Urban Q2 | 544 (18.1) | 499 (16.6) | 0.04 | 390 (15.1) | 388 (15.1) | 0 |
| Urban Q3 | 432 (14.4) | 472 (15.7) | 0.04 | 393 (15.3) | 375 (14.6) | 0.02 |
| Urban Q4 | 378 (12.6) | 384 (12.8) | 0.01 | 349 (13.5) | 349 (13.5) | 0 |
| Urban Q5 (highest) | 302 (10.1) | 282 (9.4) | 0.02 | 277 (10.7) | 245 (9.5) | 0.04 |
| Migrant status |  |  |  |  |  |  |
| Immigrant^a^ | 76 (2.5) | 72 (2.4) | 0.01 | 109 (4.2) | 101 (3.9) | 0.02 |
| Non-immigrant | 2,924 (97.5) | 2,928 (97.6) | 0.01 | 2,468 (95.8) | 2,476 (96.1) | 0.02 |
| **Smoking characteristics** |  |  |  |  |  |  |
| Frequency of smoking |  |  |  |  |  |  |
| Daily | **2,939 (98.0)** | **2,722 (90.7)** | **0.32** | **2,530 (98.2)** | **2,394 (92.9)** | **0.26** |
| Occasional | **61 (2.0)** | **278 (9.3)** | **0.32** | **47 (1.8)** | **183 (7.1)** | **0.26** |
| Cigarettes per day, mean ± SD | 15.90 ± 8.40 | 15.08 ± 8.23 | 0.1 | 19.25 ± 9.95 | 19.25 ± 9.80 | 0 |
| Age first tried smoking, mean ± SD | 15.52 ± 4.38 | 15.38 ± 3.98 | 0.03 | 15.59 ± 4.38 | 15.36 ± 4.05 | 0.05 |
| Duration smoking (years), mean ± SD | 26.28 ± 12.66 | 26.44 ± 12.56 | 0.01 | 26.71 ± 13.34 | 26.99 ± 12.99 | 0.02 |
| **Health comorbidities** |  |  |  |  |  |  |
| Prevalent comorbidities |  |  |  |  |  |  |
| COPD | **582 (19.4)** | **347 (11.6)** | **0.22** | **462 (17.9)** | **254 (9.9)** | **0.23** |
| Hypertension | 0 | 0 | 0 | 0 | 0 | 0 |
| Diabetes | **233 (7.8)** | **151 (5.0)** | **0.11** | **238 (9.2)** | **143 (5.5)** | **0.14** |
| Asthma | **742 (24.7)** | **573 (19.1)** | **0.14** | 352 (13.7) | 287 (11.1) | 0.08 |
| Cancer | 69 (2.3) | 74 (2.5) | 0.01 | 57 (2.2) | 57 (2.2) | 0 |
| Myocardial infarction | 15 (0.5) | ≤ 5 (0.2) | 0.06 | 46 (1.8) | 21 (0.8) | 0.09 |
| Congestive heart failure | 15 (0.5) | 8 (0.3) | 0.04 | 21 (0.8) | 9 (0.3) | 0.06 |
| No. ADG comorbidities, mean ± SD |  |  |  |  |  |  |
| 0-5 | 1,510 (50.3) | 1,366 (45.5) | 0.1 | **1,746 (67.8)** | **1,898 (73.7)** | **0.13** |
| 6-9 | 1,099 (36.6) | 1,182 (39.4) | 0.06 | 640 (24.8) | 553 (21.5) | 0.08 |
| 10+ | 391 (13.0) | 452 (15.1) | 0.06 | **191 (7.4)** | **126 (4.9)** | **0.11** |
| **Healthcare service use^b^** |  |  |  |  |  |  |
| Outpatient visits |  |  |  |  |  |  |
| Any outpatient visit | **2,880 (96.0)** | **2,772 (92.4)** | **0.15** | **2,364 (91.7)** | **2,035 (79.0)** | **0.37** |
| Mean ± SD rate ppy | 6.49 ± 8.47 | 6.40 ± 7.85 | 0.01 | **5.14 ± 8.95** | **3.93 ± 7.46** | **0.15** |
| ED visits |  |  |  |  |  |  |
| Any ED visit | 1,832 (61.1) | 1,718 (57.3) | 0.08 | **1,527 (59.3)** | **1,322 (51.3)** | **0.16** |
| Mean ± SD rate ppy | 0.97 ± 1.53 | 0.97 ± 1.62 | 0.01 | 0.80 ± 1.16 | 0.71 ± 1.29 | 0.07 |
| Hospitalizations |  |  |  |  |  |  |
| Any hospitalization | 461 (15.4) | 525 (17.5) | 0.06 | 277 (10.7) | 238 (9.2) | 0.05 |
| Mean ± SD rate ppy | 0.11 ± 0.32 | 0.12 ± 0.33 | 0.05 | 0.08 ± 0.27 | 0.07 ± 0.26 | 0.04 |

Note. Number (%) are reported unless otherwise noted. **Bolded SMD values are > 0.1 and indicate imbalance between cohorts.** Abbreviations: ADG = Aggregated Diagnostic Groups; COPD = chronic obstructive pulmonary disease; SD = standard deviation; ppy = per person year; ED = emergency department; Q = quintile; IQR = interquartile range; SMD = standardized mean difference.

^a^ Includes immigrants and refugees.

^b^ During 2 year period up to index date.
