# Supplementary material for: Incidence of chronic disease following smoking cessation treatment: A matched cohort study using linked administrative healthcare data in Ontario, Canada
Source: PLoS One. 2023 Jul 26;18(7):e0288759. doi: 10.1371/journal.pone.0288759 (PMC10370896; doi:10.1371/journal.pone.0288759)
Supplement: S5 Table — (DOCX) [file pone.0288759.s007.docx]

**S5 Table.** Baseline characteristics of matched treatment and control females and males, at risk for major cardiovascular events at index date

|  | **Female** | | | **Male** | | |
| --- | --- | --- | --- | --- | --- | --- |
|  | Treatment cohort  (n=5,007) | Control  cohort  (n=5,007) | SMD | Treatment cohort  (n=4,272) | Control  cohort  (n=4,272) | SMD |
| **Sociodemographic characteristics** |  |  |  |  |  |  |
| Age, mean ± SD | 48.13 ± 14.23 | 48.16 ± 14.24 | 0 | 48.16 ± 13.97 | 48.19 ± 13.94 | 0 |
| Education quintile |  |  |  |  |  |  |
| Missing | 264 (5.3) | 265 (5.3) | 0 | 257 (6.0) | 247 (5.8) | 0.01 |
| Q1 (lowest) | 333 (6.7) | 331 (6.6) | 0 | 324 (7.6) | 281 (6.6) | 0.04 |
| Q2 | 760 (15.2) | 727 (14.5) | 0.02 | 616 (14.4) | 642 (15.0) | 0.02 |
| Q3 | 973 (19.4) | 998 (19.9) | 0.01 | 894 (20.9) | 842 (19.7) | 0.03 |
| Q4 | 1,275 (25.5) | 1,278 (25.5) | 0 | 1,080 (25.3) | 1,085 (25.4) | 0 |
| Q5 (highest) | 1,402 (28.0) | 1,408 (28.1) | 0 | 1,101 (25.8) | 1,175 (27.5) | 0.04 |
| Employment quintile |  |  |  |  |  |  |
| Missing | 264 (5.3) | 265 (5.3) | 0 | 257 (6.0) | 247 (5.8) | 0.01 |
| Q1 (lowest) | 1,102 (22.0) | 1,122 (22.4) | 0.01 | 935 (21.9) | 954 (22.3) | 0.01 |
| Q2 | 1,014 (20.3) | 933 (18.6) | 0.04 | 855 (20.0) | 807 (18.9) | 0.03 |
| Q3 | 862 (17.2) | 975 (19.5) | 0.06 | 816 (19.1) | 823 (19.3) | 0 |
| Q4 | 905 (18.1) | 902 (18.0) | 0 | 730 (17.1) | 733 (17.2) | 0 |
| Q5 (highest) | 860 (17.2) | 810 (16.2) | 0.03 | 679 (15.9) | 708 (16.6) | 0.02 |
| Rurality + neighbourhood income quintile |  |  |  |  |  |  |
| Missing | 10 (0.2) | 13 (0.3) | 0.01 | 7 (0.2) | ≤ 5 (0.1) | 0.01 |
| Rural | 1,151 (23.0) | 1,180 (23.6) | 0.01 | 1,037 (24.3) | 1,040 (24.3) | 0 |
| Urban Q1 (lowest) | 1,108 (22.1) | 1,093 (21.8) | 0.01 | 931 (21.8) | 914 (21.4) | 0.01 |
| Urban Q2 | 850 (17.0) | 842 (16.8) | 0 | 637 (14.9) | 659 (15.4) | 0.01 |
| Urban Q3 | 723 (14.4) | 753 (15.0) | 0.02 | 629 (14.7) | 642 (15.0) | 0.01 |
| Urban Q4 | 670 (13.4) | 641 (12.8) | 0.02 | 569 (13.3) | 568 (13.3) | 0 |
| Urban Q5 (highest) | 495 (9.9) | 485 (9.7) | 0.01 | 462 (10.8) | 444 (10.4) | 0.01 |
| Migrant status |  |  |  |  |  |  |
| Immigrant^a^ | 104 (2.1) | 103 (2.1) | 0 | 145 (3.4) | 157 (3.7) | 0.02 |
| Non-immigrant | 4,903 (97.9) | 4,904 (97.9) | 0 | 4,127 (96.6) | 4,115 (96.3) | 0.02 |
| **Smoking characteristics** |  |  |  |  |  |  |
| Frequency of smoking |  |  |  |  |  |  |
| Daily | **4,907 (98.0)** | **4,565 (91.2)** | **0.31** | **4,188 (98.0)** | **3,955 (92.6)** | **0.26** |
| Occasional | **100 (2.0)** | **442 (8.8)** | **0.31** | **84 (2.0)** | **317 (7.4)** | **0.26** |
| Cigarettes per day, mean ± SD | 16.41 ± 8.98 | 15.68 ± 8.56 | 0.08 | 19.35 ± 10.15 | 19.50 ± 10.11 | 0.01 |
| Age first tried smoking, mean ± SD | 15.93 ± 4.75 | 15.93 ± 4.71 | 0 | 15.67 ± 4.74 | 15.37 ± 4.44 | 0.07 |
| Duration smoking (years), mean ± SD | 32.19 ± 14.01 | 32.23 ± 13.81 | 0 | 32.49 ± 14.65 | 32.81 ± 14.42 | 0.02 |
| **Health comorbidities** |  |  |  |  |  |  |
| Prevalent comorbidities |  |  |  |  |  |  |
| COPD | **1,512 (30.2)** | **1,016 (20.3)** | **0.23** | **1,118 (26.2)** | **743 (17.4)** | **0.21** |
| Hypertension | 1,272 (25.4) | 1,291 (25.8) | 0.01 | 1,164 (27.2) | 1,029 (24.1) | 0.07 |
| Diabetes | 646 (12.9) | 512 (10.2) | 0.08 | **670 (15.7)** | **461 (10.8)** | **0.14** |
| Asthma | **1,204 (24.0)** | **939 (18.8)** | **0.13** | 562 (13.2) | 454 (10.6) | 0.08 |
| Cancer | 203 (4.1) | 183 (3.7) | 0.02 | 179 (4.2) | 165 (3.9) | 0.02 |
| Myocardial infarction | 39 (0.8) | 28 (0.6) | 0.03 | 88 (2.1) | 86 (2.0) | 0 |
| Congestive heart failure | 60 (1.2) | 51 (1.0) | 0.02 | 65 (1.5) | 53 (1.2) | 0.02 |
| No. ADG comorbidities, mean ± SD |  |  |  |  |  |  |
| 0-5 | 2,315 (46.2) | 2,137 (42.7) | 0.07 | 2,630 (61.6) | 2,790 (65.3) | 0.08 |
| 6-9 | 1,839 (36.7) | 1,979 (39.5) | 0.06 | 1,200 (28.1) | 1,124 (26.3) | 0.04 |
| 10+ | 853 (17.0) | 891 (17.8) | 0.02 | 442 (10.3) | 358 (8.4) | 0.07 |
| **Healthcare service use^b^** |  |  |  |  |  |  |
| Outpatient visits |  |  |  |  |  |  |
| Any outpatient visit | **4,837 (96.6)** | **4,701 (93.9)** | **0.13** | **4,009 (93.8)** | **3,580 (83.8)** | **0.32** |
| Mean ± SD rate ppy | 7.02 ± 8.54 | 7.04 ± 7.85 | 0 | **5.80 ± 8.63** | **4.86 ± 7.28** | **0.12** |
| ED visits |  |  |  |  |  |  |
| Any ED visit | 2,973 (59.4) | 2,750 (54.9) | 0.09 | **2,472 (57.9)** | **2,145 (50.2)** | **0.15** |
| Mean ± SD rate ppy | 0.92 ± 1.44 | 0.89 ± 1.54 | 0.02 | 0.81 ± 1.31 | 0.72 ± 1.38 | 0.07 |
| Hospitalizations |  |  |  |  |  |  |
| Any hospitalization | 791 (15.8) | 848 (16.9) | 0.03 | 537 (12.6) | 457 (10.7) | 0.06 |
| Mean ± SD rate ppy | 0.11 ± 0.33 | 0.13 ± 0.35 | 0.03 | 0.09 ± 0.33 | 0.08 ± 0.30 | 0.04 |

Note. Number (%) are reported unless otherwise noted. **Bolded SMD values are > 0.1 and indicate imbalance between cohorts.** Abbreviations: ADG = Aggregated Diagnostic Groups; COPD = chronic obstructive pulmonary disease; SD = standard deviation; ppy = per person year; ED = emergency department; Q = quintile; IQR = interquartile range; SMD = standardized mean difference.

^a^ Includes immigrants and refugees.

^b^ During 2 year period up to index date.
